# Three-dimensional structure of a *Streptomyces sviceus* GNAT acetyltransferase with similarity to the C-terminal domain of the human GH84 *O*-GlcNAcase

**DOI:** 10.1107/S1399004713029155

**Published:** 2013-12-31

**Authors:** Yuan He, Christian Roth, Johan P. Turkenburg, Gideon J. Davies

**Affiliations:** a College of Chemistry and Materials Science, Northwest University, Xi’an 710069, People’s Republic of China; b York Structural Biology Laboratory, Department of Chemistry, The University of York, York YO10 5DD, England

**Keywords:** acetyltransferases, *O*-GlcNAc, GCN5, GNAT, acetyl-CoA

## Abstract

The mammalian *O*-GlcNAc hydrolysing enzyme *O*-GlcNAcase (OGA) is a multi-domain protein with glycoside hydrolase activity in the N-terminus and with a C-terminal domain that has low sequence similarity to known acetyltransferases, prompting speculation, albeit controversial, that the C-terminal domain may function as a histone acetyltransferase (HAT). There are currently scarce data available regarding the structure and function of this C-terminal region. Here, a bacterial homologue of the human OGA C-terminal domain, an acetyltransferase protein (accession No. ZP_05014886) from *Streptomyces sviceus* (SsAT), was cloned and its crystal structure was solved to high resolution. The structure reveals a conserved protein core that has considerable structural homology to the acetyl-CoA (AcCoA) binding site of GCN5-related acetyltransferases (GNATs). Calorimetric data further confirm that SsAT is indeed able to bind AcCoA in solution with micromolar affinity. Detailed structural analysis provided insight into the binding of AcCoA. An acceptor-binding cavity was identified, indicating that the physiological substrate of SsAT may be a small molecule. Consistent with recently published work, the SsAT structure further questions a HAT function for the human OGA domain.

## Abstract 2

The crystal structure of a bacterial acetyltransferase with 27% sequence identity to the C-terminal domain of human *O*-GlcNAcase has been solved at 1.5 Å resolution. This *S. sviceus* protein is compared with known GCN5-related acetyltransferases, adding to the diversity observed in this superfamily.

## 1. Introduction   

The addition of O-linked β-*N*-acetylglucosamine (GlcNAc) to serine/threonine residues of nuclear and cytoplasmic proteins, known as *O*-GlcNAc modification (Torres & Hart, 1984 ▶), is an essential post-translational modification in higher eukaryotes. To date, hundreds of proteins in the nuclear and cytoplasmic compartments have been reported to be *O*-­GlcNAc modified (reviewed in Hart *et al.*, 2007 ▶). *O*-GlcNAc is a reversible dynamic modification that responds to several physiological stimuli, such as diet. The *O*-GlcNAc modification shares many similarities with protein phosphorylation and indeed is at least partially reciprocal to phosphorylation (Wang *et al.*, 2010 ▶; Hu *et al.*, 2010 ▶). Increasing evidence points towards a role for *O*-­GlcNAc modification in transcriptional regulation, which was initially proposed on the basis of RNA polymerase and transcription factor modification (Jackson & Tjian, 1988 ▶). More recent work has shown that *O*-GlcNAc modification plays a role in the repression of gene expression by polycomb group proteins (Sinclair *et al.*, 2009 ▶; Gambetta *et al.*, 2009 ▶), further linking the modification through to epi­genetic modulation of gene expression (Sakabe & Hart, 2010 ▶; reviewed by Sakabe *et al.*, 2010 ▶).

In mammalian cells, the level of *O*-GlcNAc modification is regulated by the action of two enzymes: *O*-GlcNAc transferase (OGT) is the enzyme that is responsible for the addition of *O*-GlcNAc from uridine diphosphate *N*-acetylglucosamine to Ser/Thr of proteins, while *O*-GlcNAcase (OGA) is the glycoside hydrolase that specifically catalyses the cleavage of GlcNAc from modified proteins (Dong & Hart, 1994 ▶). Full-length human OGA (hOGA) belongs to glycoside hydrolase family 84 (GH84) in the CAZy classification of carbohydrate-active enzymes (Cantarel *et al.*, 2009 ▶) and is a 916-amino-acid protein with two distinctive structural domains linked by a region that is sensitive to caspase-3 cleavage (Butkinaree *et al.*, 2008 ▶). The *O*-GlcNAcase activity lies in the N-terminal domain (residues 60–366), the mechanism of which has been extensively studied (see, for example, Macauley, Whitworth *et al.*, 2005 ▶; Macauley, Stubbs *et al.*, 2005 ▶; He *et al.*, 2010 ▶). At the structural level, several structures of bacterial homologues of the glycoside hydrolase domain have been determined (see, for example, Rao *et al.*, 2006 ▶; Schimpl *et al.*, 2010 ▶; Dennis *et al.*, 2006 ▶) and these have been beneficial in the design and analysis of *O*-­GlcNAcase inhibitors, notably in the therapeutic context (see, for example, Yuzwa *et al.*, 2008 ▶). In contrast, the C-­terminal domain of hOGA (approximately residues 583–916) is less well characterized. Earlier bioinformatics studies (Comtesse *et al.*, 2001 ▶; Schultz & Pils, 2002 ▶) predicted that the C-terminus of hOGA had similar structural motifs to acetyltransferases. In a later biochemical study, Toleman *et al.* (2004 ▶) proposed that the C-­terminal domain of hOGA has intrinsic histone acetyltransferase (HAT) activity *in vitro*.

Initially motivated by the proposed role of the C-terminal domain of hOGA as a putative histone acetyltransferase, we sought to provide preliminary molecular insights into the possible biological function of the C-terminus of hOGA through the solution of the three-dimensional structure of a bacterial homologue. During the course of this work, however, questions arose about whether the hOGA C-terminal domain is a histone acetyltransferase, at least when acting in isolation; other groups have failed to repeat past observations (Butkinaree *et al.*, 2008 ▶). Here, we present the three-dimensional structure of a bacterial homologue of the hOGA C-terminal domain which provides no support for a role of the hOGA C-­terminal domain as a histone acetyltransferase. The acetyltransferase (accession No. ZP_05014886) from *Streptomyces sviceus* (SsAT), which shares ∼27% sequence identity with the C-terminal domain of hOGA, was cloned and the structure was determined at 1.5 Å resolution using an Hg derivative for phasing. We present the three-dimensional X-­ray crystal structure of SsAT and a comparison with related GNAT-family acetyltransferases and their complexes, providing insight into AcCoA binding and highlighting key differences from the hOGA C-terminal domain.

## 2. Materials and methods   

### 2.1. Gene cloning   

Genomic DNA of *S. sviceus* ATCC 29083 was isolated from a 0.5 ml DSM 924 bacterial cell culture using the DNeasy Blood & Tissue Kit (Qiagen). The gene encoding the acetyltransferase (ZP_05014886), termed SsAT, was amplified using the polymerase chain reaction (PCR) from *S. sviceus* genomic DNA. The forward primer had the sequence 5′-CCAGG­GACCAGCAATGTACGGGGCACGCGACCG-3′ and the reverse primer had the sequence 5′-GAGGAGAAGGCG­CGTTATCACCGGCCTTCTTCTGCCGTG-3′. The amplification product was cleaned using a QIAquick Gel Extraction Kit (Qiagen) and was cloned following a ligation-independent cloning (LIC) method (Bonsor *et al.*, 2006 ▶; Fogg & Wilkinson, 2008 ▶) into the vector pETYSBLIC-3C, which incorporates a 3C protease-cleavable N-terminal hexahistidine tag.

### 2.2. Gene expression and protein purification   

The N-terminally His-tagged SsAT plasmid was transformed into *Escherichia coli* BL21 (DE3) competent cells for expression. Transformants were plated onto LB (Luria–Bertani) agar containing 40 µg ml^−1^ kanamycin and incubated overnight at 37°C. A few colonies from this plate were used to inoculate liquid LB medium (supplemented with 40 µg ml^−1^ kanamycin). The cell culture was then grown at 37°C with shaking until an OD_600_ of 0.6 was reached, whereupon the temperature was reduced to 16°C and 1 m*M* isopropyl β-d-1-thiogalactopyranoside (IPTG) was introduced to initiate gene overexpression. To purify SsAT for crystallization, the overnight culture was spun down and the cells were subsequently lysed by sonication in lysis buffer (20 m*M* HEPES pH 7.5, 200 m*M* sodium chloride, 20 m*M* imidazole) in the presence of protease inhibitors (cOmplete Protease Inhibitor Cocktail Tablet, EDTA-free). After centrifugation to obtain a soluble cell-free extract, the SsAT protein was purified to homogeneity using Ni-affinity chromatography followed by gel filtration using a Superdex 75 FPLC column (pre-equilibrated in 20 m*M* HEPES pH 7.5, 300 m*M* sodium chloride). Fractions containing pure recombinant protein were pooled together and concentrated to ∼12 mg ml^−1^ prior to crystallization.

### 2.3. SEC–MALLS   

Analytical size-exclusion chromatography coupled to multi-angle laser light scattering (SEC–MALLS) was used to estimate the oligomeric state of SsAT in solution. SEC was performed using a Shimadzu HPLC system with an analytical Superdex 75 HR 10/30 column equilibrated with 20 m*M* HEPES pH 7.5, 300 m*M* NaCl. 100 µl of protein at approximately 1.4 mg ml^−1^ was loaded onto the column and eluted at a flow rate of 0.5 ml min^−1^ at 20°C. The elution profile was monitored by the absorbance at 280 nm. SEC was also coupled with a Wyatt DAWN HELEOS II 18-angle light-scattering detector and a Wyatt Optilab rEX refractive-index monitor. All data, including the UV, light-scattering and refractive-index traces, were recorded using the *ASTRA* software (Wyatt Technologies), which was used to perform data analysis and to estimate the molecular masses of the eluted peaks.

### 2.4. ITC   

Isothermal titration calorimetry (ITC) was performed using a MicroCal VC calorimeter (Northampton, Massachusetts, USA) at 25°C. Freshly purified SsAT was extensively dialysed against 50 m*M* HEPES pH 7.5, 200 m*M* NaCl and concentrated to a concentration of 97 µ*M*. The same buffer was used to prepare a 1.4 m*M* solution of the cofactor acetyl coenzyme A (AcCoA). For each titration, 10 µl aliquots of the AcCoA solution were injected into the SsAT solution in the cell with spacings of 4 min. The experimental data were fitted to a one-site binding model using the MicroCal *Origin* software, with the stoichiometry (*n*), enthalpy (Δ*H*°) and association constant (*K*_a_) as adjustable parameters.

### 2.5. Crystallization and structure solution   

The initial search for crystallization conditions was carried out in a 96-well sitting-drop format using MRC Wilden plates. Commercially available screens manufactured by Hampton Research (USA) as well as tailor-made screens were used to find suitable starting conditions. Drops of 0.3 µl total volume with a protein:reservoir ratio of 1:1 were set up over 54 µl reservoir solution using a Mosquito robot. Initial starting conditions were found in the Index as well as the PACT screen with PEGs of different size as precipitant. Optimization of the initial conditions was performed in a 24-well hanging-drop format with a total drop volume of 2 µl. The crystals used for data collection were obtained from mother liquor comprising 0.2 *M* MgCl_2_, 0.1 *M* imidazole pH 8.0, 25%(*w*/*v*) PEG 3350 at 293 K. Crystals grew within two weeks to a maximum size of approximately 150 × 50 × 50 µm. Before data collection, a single crystal was cryoprotected in well solution supplemented with 20%(*v*/*v*) glycerol and subsequently flash-cooled in liquid nitrogen. A native data set was collected to 1.5 Å resolution at 100 K on beamline ID14-1 of the European Synchrotron Radiation Facility (ESRF), Grenoble equipped with an ADSC Quantum 210 CCD detector. The data were collected with an rotation range of 0.5° per frame over a total rotation range of 180°.

For initial phasing, a mercury heavy-atom derivative was prepared by overnight soaking of SsAT crystals in mother liquor containing approximately 3 m*M* mercury acetate. Single-wavelength diffraction data for the mercury acetate-derived crystal were collected in-house at 120 K to a resolution of 2.75 Å using a Cu *K*α rotating-anode generator (Rigaku MicroMax) and a MAR345 image-plate detector. Data were collected over 360° with 0.5° rotation per frame.

Both the native and the derivative data were integrated using *MOSFLM* (Leslie, 1992 ▶) and reduced and scaled using *SCALA* from the *CCP*4 suite of programs (Winn *et al.*, 2011 ▶). Scaling the amplitudes of the derivative data to the native data set gave an *R*_isom_ of 0.193. Using the SIRAS (single isomorphous replacement with anomalous scattering) method in the *SHELXC*/*D*/*E* suite of programs (Sheldrick, 2008 ▶), as implemented in *CCP*4*i*, the heavy-atom site was readily located and phasing from the SIRAS data was performed using a solvent fraction of 45%. The solvent–protein contrast, reflected in the standard deviation of the local r.m.s. electron density, calculated by *SHELXE* indicated the correct enantiomorph unambiguously. The resulting phases from *SHELXE* were used in automated model building and refinement with *ARP*/*wARP* (Perrakis *et al.*, 1997 ▶). Approximately 70% of the model was built using this software package, and this initial model was further improved by alternating cycles of manual model building in *Coot* (Emsley & Cowtan, 2004 ▶) associated with refinement in *REFMAC* (Murshudov *et al.*, 2011 ▶). The quality of the final structure was validated using *MolProbity* (Chen *et al.*, 2010 ▶).

The coordinates and structure factors of SsAT have been deposited in the Protein Data Bank as entry 4bmh.

## 3. Results   

### 3.1. Protein production   

The recombinant SsAT protein could be produced in a soluble form in *E. coli*. SsAT appears to be a monomer in solution, as the estimated molecular weight from the SEC–MALLS experiment agrees well with the calculated molecular weight of 29 kDa based on the amino-acid sequence. SDS gels (data not shown) of purified SsAT showed that this protein was subject to degradation over time. The apparent molecular weight of the degraded SsAT protein appeared to be 24 kDa (5 kDa smaller than the full-length protein) as judged from SDS gels and SEC–MALLS.

### 3.2. Structure solution and three-dimensional structure of SsAT   

**Figure 1:**
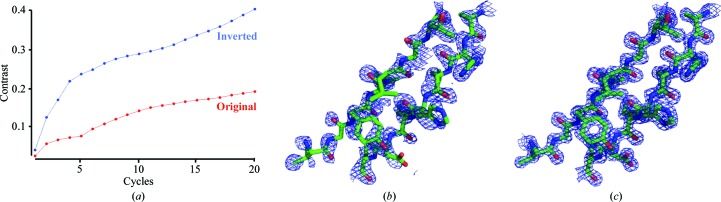
SIRAS phasing of SsAT. (*a*) Progress of density modification, monitored by the protein–solvent contrast *versus* modification cycles, using *SHELXE*. (*b*) Selective region of electron density in the solvent-flattened map with experimental phases. (*c*) Electron-density map (2*F*_o_ − *F*_c_) of the same region in the model after automatic residue building in *ARP*/*wARP*. All maps are contoured at 1σ.

The SsAT protein crystallized in space group *P*3_2_21, with unit-cell parameters *a* = *b* = 75.9, *c* = 76.5 Å, α = β = 90, γ = 120°, and diffracted to 1.5 Å resolution (Table 1 ▶). Initial attempts to solve the phase problem by molecular replacement using available acetyltransferase models were unsuccessful. Therefore, experimental phasing with a single heavy-atom derivative was carried out using the SIRAS method. A single mercury ion, which was bound to the only cysteine in SsAT, was sufficient for phasing. The correct solution could readily be identified after density modification by manual inspection of the initial electron-density maps (Fig. 1 ▶). The final model, with an *R*_cryst_ and an *R*_free_ of 0.15 and 0.17, respectively, is comprised of 198 amino acids (residues 56–245 and 248–259), one chloride ion and 300 water molecules. The final crystallized construct corresponds to the truncated variant as a result of the degradation of the protein.

**Figure 2:**
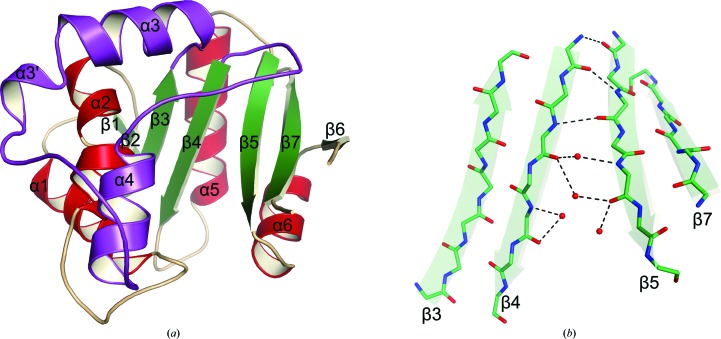
Three-dimensional crystal structure of SsAT. (*a*) The overall architecture of SsAT. The secondary-structure elements are shown in different colours. (*b*) Close-up view of the four central β-strands in SsAT, highlighting the characteristic splay between β4 and β5 in the structure. Water molecules are shown as red spheres. Hydrogen-bond interactions are shown as blue dashed lines.

SsAT is a globular protein and the overall structure displays a mixed α/β-fold with a central seven-stranded β-sheet surrounded by eight α-helices (Fig. 2 ▶ *a*). The most notable feature of the structure is that the two central parallel strands (β4 and β5) splay out in the middle, dividing the whole structure into two parts (Fig. 2 ▶ *b*). The resulting cleft is stabilized by a network of water molecules, which make extensive hydrogen-bond interactions with the main-chain amide and carbonyl O atoms of residues from both β4 and β5. This splayed structure has previously been characterized as a unique feature of the GCN5-related *N*-acetyltransferases (GNATs; Vetting *et al.*, 2005 ▶).

### 3.3. ITC analysis of AcCoA binding   

**Figure 3:**
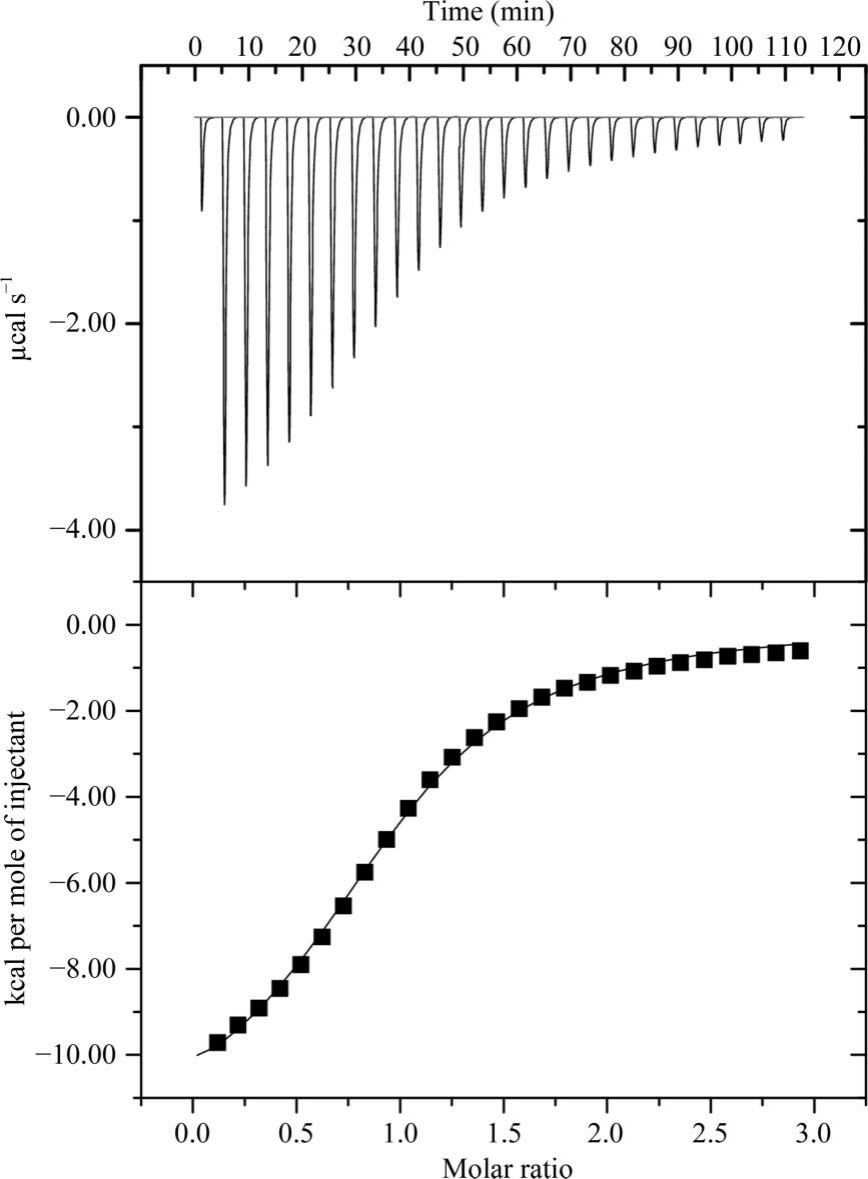
ITC titration of SsAT with AcCoA (pH 7.5, 25°C). The top panel shows the raw data for the titration of the ligand against the protein. The lower panel shows the fit of the data assuming a bimolecular binding model.

The universal activity of GNATs is to catalyse the transfer of an acetyl group from the cofactor acetyl coenzyme A (AcCoA) to a primary amine on the acceptor. The binding of AcCoA to SsAT was determined by isothermal titration calorimetry at pH 7.5 and 25°C (Fig. 3 ▶). The data revealed that AcCoA can bind to SsAT with a dissociation constant (*K*_d_) of 20 µ*M* under the conditions used. The stoichiometric value derived from the titration curve is close to 1, indicating a one-­to-one binding between SsAT and the cofactor. Thermodynamically, association of SsAT with AcCoA is exothermic and is driven by a large favourable binding enthalpy (Δ*H* = −12.4 kcal mol^−1^), which is partially offset by an unfavourable binding entropy (*T*Δ*S* = −5.9 kcal mol^−1^). However, we were unable to crystallize a complex of SsAT with AcCoA.

## 4. Discussion   

Human *O*-GlcNAcase (hOGA) is a protein whose C-terminus has been reported to have putative histone acetyltransferase (HAT) activity. In this study, a bacterial homologue of the C-­terminus of hOGA from *S. sviceus* (SsAT) was solved at a resolution of 1.5 Å using the SIRAS method. Electron density was observed for most of the residues, with the exception of the N-­terminal His tag and the first 30 amino acids in the sequence of SsAT, reflecting degradation of SsAT over the course of storage and subsequent crystallization. Multi-sequence alignment of SsAT with its close homologues reveals that the N-­terminal region of some 30 amino acids in SsAT is poorly conserved among *Streptomyces*. This N-terminal region may therefore be intrinsically unstable. Interestingly, a functionally related enzyme (sheep serotonin *N*-acetyltransferase) has also been reported to have a particularly protease-sensitive N-­terminus (Hickman *et al.*, 1999 ▶).

### 4.1. Structural similarity searches   

Structural homology searches performed using two individual online servers, *SSM*/*PDBeFold* (http://www.ebi.ac.uk/msd-srv/ssm; Krissinel & Henrick, 2004 ▶) and *ProFunc* (Laskowski *et al.*, 2005 ▶), indeed match SsAT to known acetyltransferases. The closest structure to SsAT obtained using *PDBeFold* was the acetyltransferase domain of the NATA N-­terminal acetyltransferase from *Schizosaccharomyces pombe* (PDB entry 4kvm; 21% sequence identity, *Z*-score of 10.9, with 153 C^α^ atoms overlapping with an r.m.s.d. of 1.8 Å; Liszczak *et al.*, 2013 ▶). Many other GCN5-related acetyltransferases (GNATs) overlap with similar values (for example, with *Z*-scores of >10), including the aminoglycoside acetyltransferase AAC(6′)-Ii from *Enterococcus faecium* (PDB entry 2a4n; r.m.s.d. of 1.8 Å over 130 C^α^ atoms, 14% sequence identity; Burk *et al.*, 2005 ▶) and serotonin *N*-acetyltransferase from *Ovis aries* (PDB entry 1kuy; r.m.s.d. of 1.9 Å over 124 C^α^ atoms, 23% sequence identity; Wolf *et al.*, 2002 ▶).

GNAT is a superfamily of enzymes whose activity is to catalyse the transfer of an acetyl group from the cofactor acetyl coenzyme A (AcCoA) to the primary amines of a diverse set of acceptors ranging from small molecules (such as aminoglycoside antibiotics and arylalkylamines) to large proteins (such as ribosomes and histones) (Dyda *et al.*, 2000 ▶; Vetting *et al.*, 2005 ▶).

### 4.2. SsAT and the GNAT superfamily   

**Figure 4:**
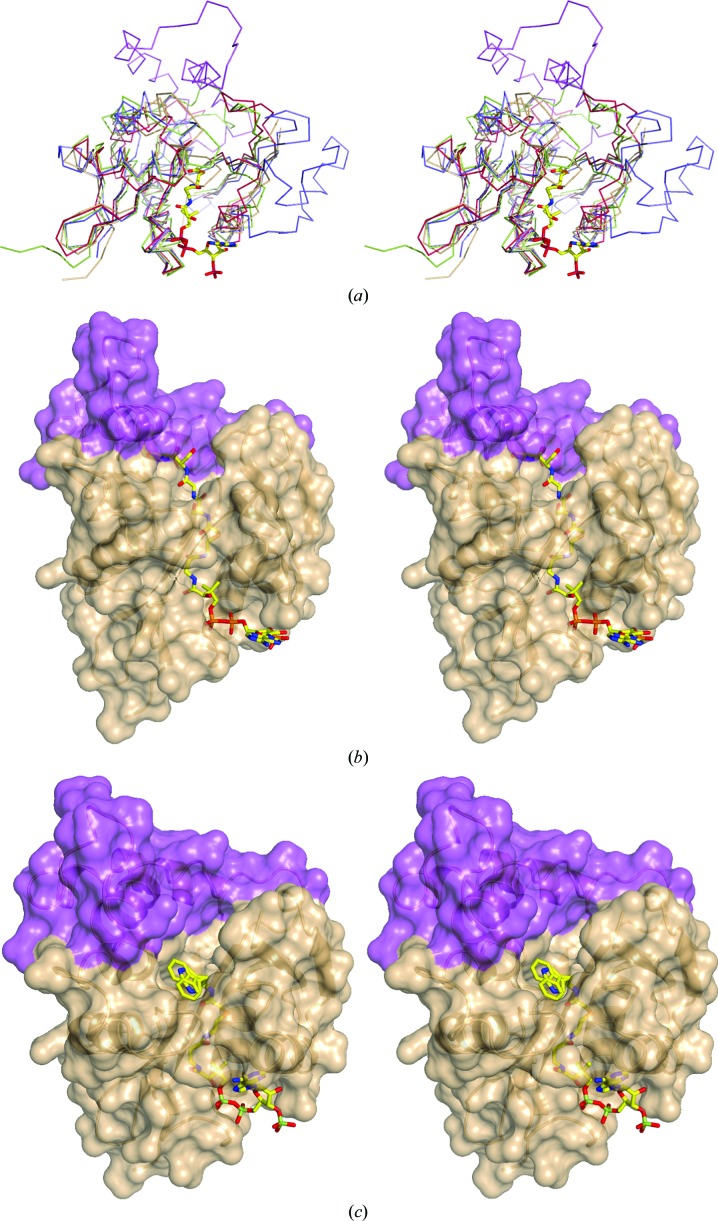
Structural comparison of different acetyltransferases. (*a*) Superposition of the backbone of SsAT (fawn) with the 55-residue insertion (purple) with NATA (PDB entry 4kvm, dark red), serotonin *N*-­acetyltransferase AANAT (PDB entry 1kuy, green) and aminoglycoside acetyltransferase AAC (PDB entry 2a4n, blue). The AcCoA moiety based on the NATA complex is shown in stick representation to mark the binding site. All structures show the same conserved core structure with the characteristic splay between β-strands 4 and 5 necessary for AcCoA binding. (*b*) Cartoon represention of SsAT with its solvent-accessible surface overlaid with the NATA bisubstrate complex (PDB entry 4kvm). The bound bisubstrate, shown in stick representation, binds with its AcCoA part in the binding site. The acceptor peptide moiety clashes with the unique 55-­amino-acid insertion of SsAT (purple), which is not present in NATA. (*c*) The cartoon represention of SsAT with its solvent-accessible surface overlaid with the serotonin *N*-acetyltransferase bisubstrate complex (PDB entry 1kuy). The bisubstrate binds within the binding groove of SsAT with the acceptor tryptamine moiety pointing into a pocket and overlays partially with the remaining unmodelled density, attributed as a possible yet unidentified acceptor.

**Figure 5:**
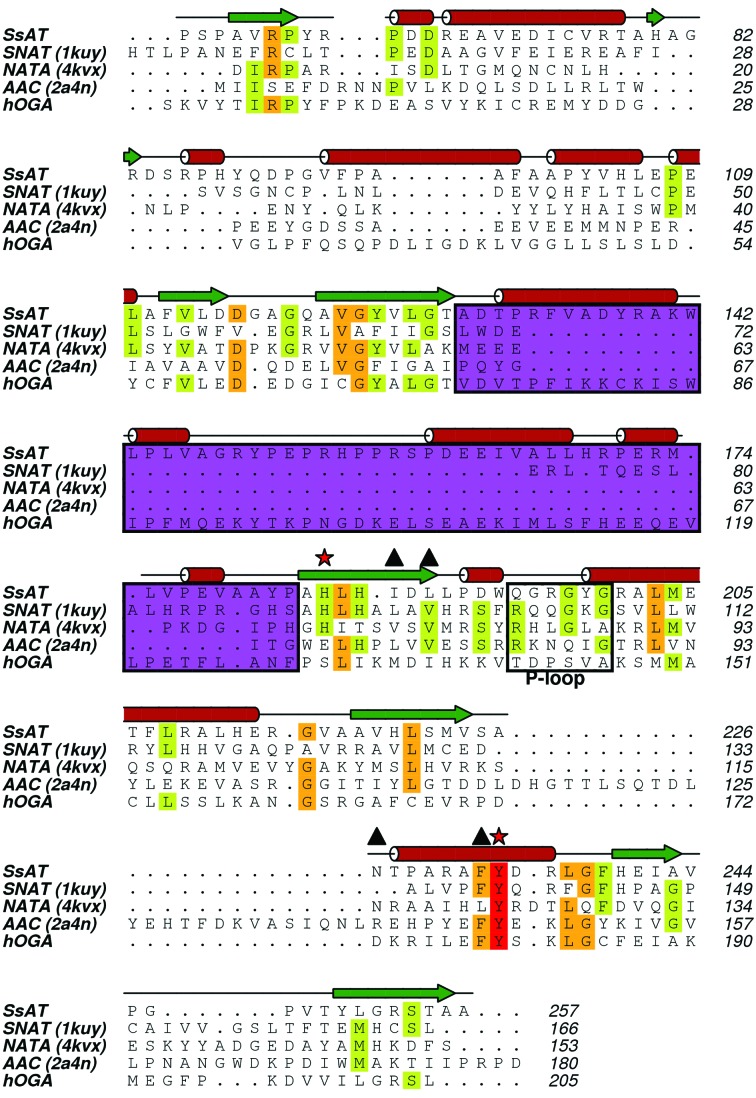
Multi-sequence alignment of SsAT, hOGA and several GNATs referenced in the text. Secondary-structural elements are highlighted, with α-helices as cylinders and β-strands as arrows, based on the secondary-structure assignment of SsAT. The P-loop responsible for the binding of the pyrophosphate moiety of AcCoA is highlighted in a box. Note that hOGA shows an ‘atypical’ sequence in this region. A tyrosine and a histidine (marked with red stars) have been proposed to act as catalytic residues. Several residues, besides the P-loop, that interact with AcCoA are marked with triangles. The region between β3 and β4 coloured in purple shows a variable length in different acetyltransferase structures; SsAT and hOGA both have unique extensive insertions here.

To date, thousands of protein sequences have been identified as members of the GNAT superfamily. It is known that subfamilies across the GNAT superfamily tend to have highly variable amino-acid sequences. It is intriguing that despite the overall low sequence similarity between SsAT and other GNATs, there is a remarkable resemblance in their core protein structures (for a structural review of GNATs, see Vetting *et al.*, 2005 ▶). As shown in Fig. 4 ▶, the central elements of SsAT (β2, β3, β4, β5 and α5) are extraordinarily conserved in other GNATs. The core structure of all of these GNATs has a distinctive splay between the central β4 and β5 strands. In most of the cases, partition of β4 and β5 is induced by structural perturbation of local hydrogen-bond patterns caused by the presence of a ‘β-bulge’ (Dyda *et al.*, 2000 ▶). Although local hydrogen-bond patterns are perturbed in SsAT, we did not observe the presence of a β-bulge here (Fig. 2 ▶). Nevertheless, the splay between β4 and β5 in SsAT creates a characteristically V-shaped opening, which is known to be the binding site of AcCoA in other GNATs. The observed maximum opening between the strands is approximately 14 Å, with an opening angle of roughly 27°. Another unique feature of SsAT is a 55-residue sequence between β3 and β4 (Figs. 4 ▶ *a* and 5 ▶). In most of the GNATs shown here [*i.e.* NATA and AAC(6′)-Ii], the β3 and β4 strands are connected *via* a 3–­5-residue turn (Figs. 4 ▶ and 5 ▶), except for the serotonin *N*-­acetyltransferase (PDB entry 1kuy), which has a 27-residue insertion. This inserted sequence in SsAT (Fig. 5 ▶) forms an extra subdomain that spans over the splayed β-sheet (Fig. 4 ▶ *a*), making the central motif of SsAT less solvent-exposed than in other GNATs. An insertion of the same length is also present in the putative HAT domain of hOGA and differentiates the C-­terminal domain of hOGA from all other known members of the GNAT superfamily.

### 4.3. Cofactor binding   

Consistent with the predicted acetyltransferase activity of SsAT, the ITC data show that SsAT is indeed able to bind acetyl-CoA in solution. The dissociation constant of SsAT and AcCoA appeared to be 20 µ*M* at pH 7.5 and 25°C, and the binding event is strongly driven by a large enthalpy change, which is partially offset by an unfavourable entropy change. These values agree well with the thermodynamic parameters for the binding of AcCoA to an aminoglycoside 6′-­*N*-acetyltransferase [AAC(6′)-Ii; Hegde *et al.*, 2002 ▶], as well as to a dopamine *N*-acetyltransferase (Dat; Cheng *et al.*, 2012 ▶). The similar observed binding modes for AcCoA in different members of the GNAT family, as well as the similar thermodynamic parameters, support the conserved binding mode for AcCoA.

**Figure 6:**
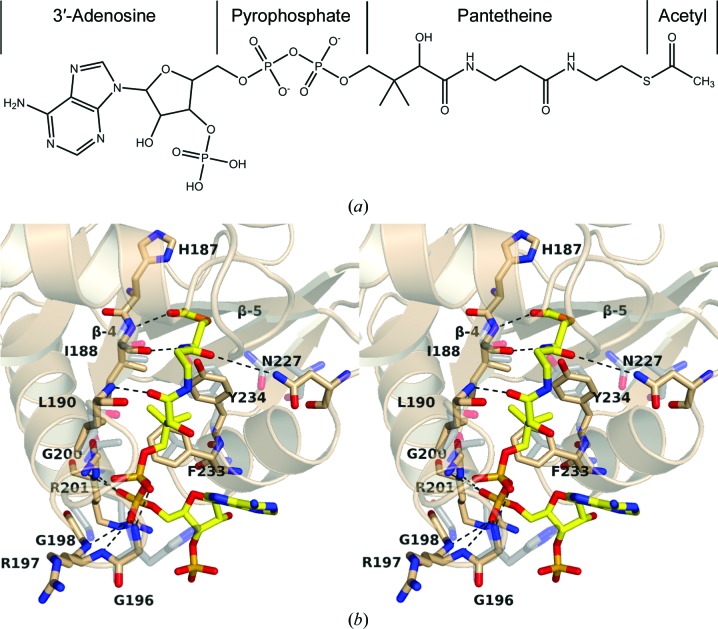
(*a*) Schematic representation of the cofactor and (*b*) a stereoview of the putative binding site for AcCo of SsATA; AcCoA is overlaid from the NATA complex (PDB entry 4kvm) in stick representation. The cavity-surrounding secondary-structure elements of SsAT are shown as a cartoon representation. Residues which are most likely to coordinate AcCoA are highlighted as sticks and are labelled accordingly. The corresponding residues in NATA (shown in grey) overlap well with the residues of SsAT, supporting the proposed binding mode. The pyrophosphate-coordinating residues correspond to the conserved P-loop, a signature motif of acetyltransferases.

Despite our unsuccessful attempts to obtain a complex structure of SsAT bound to AcCoA, it is possible to infer the binding mode of the cofactor from known complex structures. In the recent crystal structure of NATA in complex with AcCoA (Liszczak *et al.*, 2013 ▶) the cofactor is bound in an L-shaped conformation, with its pantetheine arm buried in the central cleft and the adenine and ribose ring placed on the protein surface. Interactions with NATA are predominantly established by the pantetheine moiety of AcCoA. It is interesting that among the up to eight hydrogen-bond interactions between AcCoA and NATA, four are made between the pyrophosphate moiety and the main-chain atoms on the loop following β4 (known as the P-loop; see below). This loop has a consensus sequence (Q/R-*X*-*X*-G-*X*-G/A; Neu­wald & Landsman, 1997 ▶) among all of the GNATs and it achieves coordination of the pyrophosphate moiety *via* re­arranging the main-chain atoms of several amino acids to surround the phosphate O atoms of AcCoA. The remaining hydrogen bonds are again formed exclusively between the main-chain atoms of β-strand 4 and the pantetheine arm. The predominant interactions of GNATs with their cofactor are mediated *via* main-chain atoms, which may very well reflect the virtual absence of a conserved sequence pattern between GNATs. Structural superposition of the core structure of SsAT with the NATA–AcCoA complex reveals that AcCoA could be readily placed in the active-site cleft of SsAT with no obvious steric hindrance (Fig. 6 ▶). Furthermore, all of the residues that interact with AcCoA in NATA align well with equivalent residues in SsAT. For this reason, it is tempting to speculate that AcCoA may bind SsAT in a manner similar to that observed for NATA. Based on the overlaid three-dimensional structures (Fig. 6 ▶), the pyrophosphate moiety is likely to be coordinated through the formation of five hydrogen-bond interactions with the backbone amides of Gly196, Gly200, Arg201 and Gly198. These interactions demand that AcCoA assumes a ‘bent’ conformation in order to fit into the opening created by β4 and β5.

Notably, all of these pyrophosphate-interacting amino acids are found in a loop connecting β4 and α3 known as the P-loop (Neuwald & Landsman, 1997 ▶). In agreement with SsAT as a member of the GNAT family, the sequence of the P-loop (Gln195-Gly196-Arg197-Gly198-Tyr199-Gly200) in SsAT (Fig. 5 ▶) follows the pattern observed in other GNATs. However, it is likely to be significant that the corresponding region in hOGA has the atypical sequence Thr852-Asp853-Pro854-Ser855-Val856-Ala857 (Fig. 5 ▶) compared with the GNAT family, suggesting that AcCoA may not be a substrate of hOGA. The absence of a key motif for AcCoA binding is consistent with biochemical data that show that hOGA, at least when acting alone, is not a histone acetyltransferase (Butkinaree *et al.*, 2008 ▶).

The pantetheine moiety of AcCoA would bind in an extended form (Fig. 6 ▶), with its pantetheine arm in such an orientation that it mimics the hydrogen-bonding pattern of a cognate β-strand for β4 in a β-sheet structure. The docked pantetheine moiety makes a total of three hydrogen-bond interactions. Two hydrogen bonds are established with the main-chain amide of Leu190 and the main-chain carbonyl O atom of Ile188 on β4, respectively. The third hydrogen-bond interaction is donated by the side-chain amine of Asn227 on α5, and this is likely to be the only interaction established between AcCoA and a side-chain atom of the protein. In addition to three hydrogen-bond interactions, this superposed model also indicates potential hydrophobic interactions between the dimethyl groups of AcCoA and Phe233 of SsAT. The acetyl group of AcCoA further expands into the active site, where the carbonyl of the acetyl group is hydrogen-bonded to the main-chain amide of Ile188, and the S atom of AcCoA is positioned within weak hydrogen-bonding distance of the side-chain hydroxyl group of Tyr234. It is believed (reviewed in Dyda *et al.*, 2000 ▶) that GNATs catalyse the direct transfer of the acetyl group of AcCoA, *via* nucleophilic attack at the acyl C atom by the primary amine of the acceptor, to form a tetrahedral intermediate. Direct nucleophilic attack requires the primary amine of the acceptor to be in its uncharged form. Multiple strategies to facilitate this reaction have been proposed. A conserved feature within the acetyltransferases is the conserved tyrosine, which donates a hydrogen bond to the S atom of AcCoA and may act as general acid or contribute to the proper orientation of the donor (Scheibner *et al.*, 2002 ▶). Multiple strategies are found to be used by acetyltransferases to generate the neutral amine acceptor. Many GNAT-family members have been proposed to use a structurally conserved water held in place by multiple hydrogen bonds, including important interactions with a glutamate or a histidine side chain. A candidate to fulfil this function is His187, which is semi-conserved or replaced by another polar residue. His187 may to some extent be replaced by His185, which shows a similar degree of conservation, and both together may fulfil the same role, like the His120/His122 pair in serotonin acetyltransferase (PDB entry 1kuy). A direct role of His187 as general base in conjunction with a proton relay over Tyr103 to His185 cannot be excluded at the moment. In NATA Glu24 is proposed to act directly as a catalytic base. No equivalent residue is found in SsAT which could fulfil a similar role. In the case of hOGA, the two histidines are replaced by serine and isoleucine, respectively (whilst the equivalent of Glu24 is a Phe), which does not immediately suggest the possibility of an analogous mechanism. That said, a substantial structural rearrangement was observed for NATA to reach the active state. If similar allosteric rearrangements are necessary in SsAT (or indeed hOGA), then the catalytic base would be not identifiable from the current structure.

### 4.4. The acceptor pocket of SsAT   

**Figure 7:**
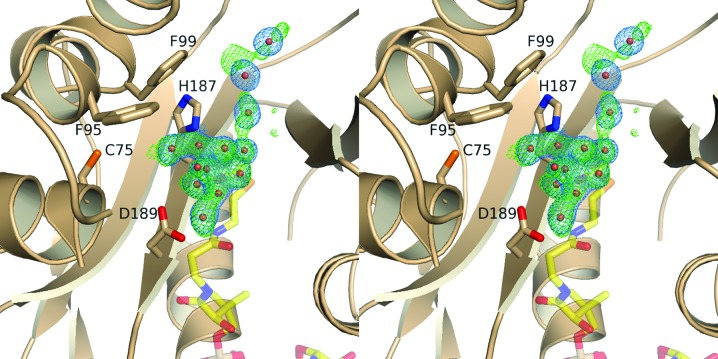
Stereoview into the putative acceptor-binding site. The *F*_o_ − *F*_c_ OMIT difference density in the active site is shown as a green mesh contoured at 2.5σ. The resulting density after refinement with dummy water atoms is shown as a blue mesh contoured at 1.0σ. Anti-bumping restraints were omitted to allow ‘covalent bonding distances’ between the dummy atoms. The residues which form the putative cavity and might interact with the acceptor are shown as sticks and are labelled accordingly. The cofactor AcCoA shown in stick representation was positioned according to the overlay with NATA (PDB entry 4kvx).

In contrast to the donor-binding site, the acceptor-binding sites of GNATs have not been well studied. There are a few structures in which bisubstrate analogues or substrate peptides were found to be bound in the structures of GNATs. Three-dimensional structure alignment of SsAT with an NATA bisubstrate complex (PDB entry 4kvm) reveals that, despite the fact that the binding mode of AcCoA is likely to be conserved between these two structures, modelling of the acceptor peptide from NATA into the SsAT structure causes severe steric clashes (Fig. 4 ▶ *b*). In contrast to the classical histone acetyltransferases, such as RimI and tGCN5 (Vetting *et al.*, 2005 ▶, 2008 ▶), with a largely solvent-exposed complementary surface groove, SsAT has a more occluded tunnel-like structure restricted mainly by the 55-residue insertion. The tunnel-like binding groove resembles the shape of the NATA substrate-binding site and may fulfil a similar role in substrate discrimination (Liszczak *et al.*, 2013 ▶). Three-dimensional alignment of SsAT with a bisubstrate complex of serotonin *N*-­acetyltransferase (PDB entry 1kuy) demonstrates that the ‘serotonin’ molecule could easily be accommodated in the putative acceptor cavity of SsAT. This observation suggests that the pocket observed here is likely to be the putative acceptor-binding site of SsAT. Interestingly, in the SsAT structure a piece of continuous density was present in this pocket which seems to extend further into the binding groove of SsAT (Fig. 7 ▶). Residues Asp189 and His187 are within hydrogen-bonding distance, of which His187 is at least partially conserved (Fig. 5 ▶). Furthermore, His187 is one candidate for the proton abstraction of the acceptor (see above), which further strengthens the assumption of a critical role for this residue in the catalytic mechanism. However, this density could not be attributed to any molecule derived from the buffer used during purification and crystallization. Whether the uninterpretable density could possibly correspond to the physiological substrate of SsAT that was picked up from the cell culture remains an open question. Nevertheless, based on the size, shape and amino-acid distribution, small polar molecules might bind to SsAT. However, elongated substrates or peptides could not be excluded as potential acceptors with certainty, as only minor conformational changes would be required to open up the acceptor site appropriately.

### 4.5. Implications for hOGA   

In the context of the comparison of SsAT with hOGA that inspired this work, the divergence away from the classical CoA-binding P-loop and the absence of a putative base, together with the potentially less exposed core structure owing to the 55-­amino-acid insertion, support the observations that the hOGA C-terminal domain is not, by itself, an acetyl-CoA-dependent histone acetyltransferase.

*Note added in proof.* During the preparation of this article for publication, the structure of another bacterial homologue of the human hOGA C-terminal putative HAT domain, that from *Oceanicola granulosus* has been published (Rao *et al.*, 2013 ▶). The *O. granulosus* acetyltransferase (OgAT), as SsAT, is clearly a GNAT superfamily acetyltransferases member and also shows the insertion between strands β3 and β4. In contrast to SsAT, Rao and colleagues successfully determined a complex of OgAT with bound AcCoA. They show that AcCoA binds in the canonical binding site between the splayed β-strands stabilized by interactions of the pyrophos­phate with the signature P-loop motif for acetyltransferases. Further, they show using surface plasmon resonance with the recombinant C-terminal putative HAT domain of hOGA, that the absence of this motive precludes binding of AcCoA. Furthermore the alteration of the OgAT P-loop sequence to make it better resemble the sequence of hOGA, abolishes AcCoA-binding in OgAT. The Rao article supports the conclusion that the hOGA C-terminal domain is unlikely to be a classical AT and the authors suggest it may thus be called a ‘pseudo-histone acetyltransferase’.

## Supplementary Material

PDB reference: SsAT, 4bmh

## Figures and Tables

**Table 1:** X-ray data-collection and structure-refinement statistics for SsAT Values in parentheses are for the highest resolution shell.

	Hg derivative†	Native
Data collection		
X-ray source	Rotating anode	ID14-1, ESRF
Wavelength (Å)	1.5418	0.9334
Space group	*P*3_2_21	*P*3_2_21
Unit-cell parameters (Å, °)	*a* = *b* = 75.5, *c* = 76.9, α = β = 90.0, γ = 120.0	*a* = *b* = 75.9, *c* = 76.5, α = β = 90.0, γ = 120.0
Resolution (Å)	77–2.75 (2.90–2.75)	65–1.50 (1.58–1.50)
*R*_merge_	0.095 (0.22)	0.086 (0.50)
*R*_meas_	0.110 (0.25)	0.089 (0.53)
*R*_p.i.m._	0.044 (0.13)	0.025 (0.16)
No. of reflections	143156 (17979)	502386 (65304)
No. of unique reflections	12637 (1740)	40822 (5853)
〈*I*/σ(*I*)〉	20.1 (9.2)	20.8 (4.9)
Completeness (%)	99.1 (93.6)	99.2 (98.3)
Multiplicity	11.3 (10.3)	12.3 (11.2)
Molecules per asymmetric unit	1	1
Matthews coefficient (Å^3^ Da^−1^)	2.63	2.65
Wilson *B* (Å^2^)	40	11
Refinement		
Resolution (Å)		20–1.5
No. of reflections		38757
*R*_cryst_/*R*_free_		0.15/0.17
No. of atoms		
Protein		1580
Water		300
*B* factors (Å^2^)		
Protein		13
Water		27
R.m.s. deviations		
Bond lengths (Å)		0.01
Bond angles (°)		1.3

† Heavy-metal phasing (one Hg site) with *SHELXD* yielded a PATFOM of 25.4, with correlation coefficients CC_all_/CC_weak_ of 28.6/20.5 and a CC_free_ for left/right-handed maps of 55.8/44.1, clearly defining the handedness of the map.
